# Defensive gun use: What can we learn from news reports?

**DOI:** 10.1186/s40621-022-00384-8

**Published:** 2022-07-01

**Authors:** David Hemenway, Chloe Shawah, Elizabeth Lites

**Affiliations:** 1 Harvard TH Chan School of Public Health, 677 Huntington Avenue, Boston, MA 02115 USA; 2 Harvard College, Cambridge, MA 02138 USA

**Keywords:** Firearms, Defensive gun use, Self-defense, Home invasions, Store robberies, Escalation arguments, News

## Abstract

**Background:**

In the past decade, most people who buy and own guns are doing so for self-defense. Yet little is known about actual defensive gun use in the USA.

**Methods:**

To discover what information newspaper articles and local news reports might add, we read the news reports of defensive use incidents assembled by the Gun Violence Archive. We examined a sample of more than a quarter of the incidents from 2019, the last year before the pandemic. We examined all cases from four months—January, April, July, and October. We created a typology of defensive gun use incidents.

**Results:**

Of 418 incidents, in about half, the perpetrator was armed with a firearm. In almost 90% of the cases, the victim fired their firearm—315 perpetrators were shot and about half of them died. The average number of perpetrators shot per incident was 0.75; the average number of victims shot was 0.25. We estimate that in 2019 fewer than 600 potential perpetrators were killed in defensive gun use incidents that made the news. Among the thirteen categories of shooting were drug-related (4% of incidents), gang-like combat (6%), romantic partner disputes (11%), escalating arguments (13%), store robberies (9%), street robberies (5%), unoccupied vehicle theft (5%), unarmed burglaries (7%), home invasions (20%), and miscellaneous (6%).

**Conclusion:**

We believe the Gun Violence Archive dataset includes the large majority of news reports of defensive gun use—and especially those in which the perpetrator is shot and dies. Some of the strengths of using news reports as a data source are that we can be certain that the incident occurred, and the reports provide us with a story behind the incident, one usually vetted in part by the police with occasional input from the victims, perpetrator, family, witnesses, or neighbors. Defensive gun use situations are quite diverse, and among the various categories of defensive gun use, a higher percentage of incidents in some of the categories seemed far less likely to be socially beneficial (e.g., drug-related, gang-like, escalating arguments) than in others (e.g., home invasions).

## Background

Little is known about defensive gun use in the USA. A review of the literature concludes that there is considerable uncertainty about both the prevalence of defensive gun use and the extent to which defensive gun use reduces harm, either for the individual defender or for society (RAND Corp 2018). Part of the problem is that self-defense is an ambiguous term, and whether one is a defender or a perpetrator in any hostile interaction may depend on perspective. The perceptions of the defender are important, but almost all of the empirical literature on defensive gun use relies solely on the survey responses of the defender (RAND Corp 2018).

The survey estimates on the annual number of defensive gun uses, provided by the two main sources of data, the federal National Crime Victimization Surveys (e.g., McDowall and Wiersema 1994) and private surveys (e.g., Kleck and Gertz 1995), differ by more than an order of magnitude. Various scholars have argued that, because of the problems of forward telescoping and the overestimating of rare events with positive desirability bias, the millions of annual defensive gun uses extrapolated from private surveys are large overestimates (Hemenway 1997; Cook et al. 1997). For example, private surveys indicate that 200,000 criminals may be shot each year by Americans who are defending themselves with firearms—an estimate which is double the number of overall firearm injuries and deaths seen in hospitals and morgues, and those victims mostly shot themselves, were shot unintentionally by someone else, or were the victims of criminal assaults (Hemenway 1997).

Studies suggest that using a gun in self-defense may not reduce injury to the defendant compared to calling the police (Tark and Kleck 2004; Hemenway and Solnick 2015), though defensive gun use may help reduce property loss (Hemenway and Solnick 2015). One study concluded that the majority of reported defensive gun uses on private surveys were probably illegal and contrary to the interests of society (Hemenway et al. 2000). But the various limitations from all research using survey data have meant that “evidence for any causal effects of defensive gun use on reducing harm to individuals or society is inconclusive” (RAND Corp 2018).

Only a few defensive gun use surveys have asked respondents to tell the story of the event (Hemenway et al. 2000; Hemenway and Azrael 2000), and these are too small to generate helpful categories of defensive gun use. None of the self-defense studies has provided a useful typology of the types of defensive gun uses.

In the current article, we examine what might be learned about defensive gun use, not from surveys, but from news reports. News reports have a long history of being used in injury prevention as an adjunct to other data sources (Sacks et al. 1992; Rainey and Runyan 1992; Stallones and Gunderson 2008; Marlenga et al. 2017). Our data source is the Gun Violence Archive (GVA 2021).

The GVA is a publicly available database of news reports (e.g., newspaper articles, local TV reports) on firearm incidents in the USA. The GVA classifies the incidents into categories such as mass shootings, unintentional shootings, and defensive use and links to news articles about the incident. The GVA has been shown to be a comprehensive source of fatal police shootings of civilians (Conner et al. 2019). A study of three cities also found it covered 83% of all fatal interpersonal shootings known to the police (Kaufman et al. 2020). The GVA is widely cited in the lay press and increasingly used in scientific research on firearm injury (Booty et al. 2019; Kim 2019; Schleimer et al. 2021; James et al. 2021; Johnson et al. 2021; Geller et al. 2021).

The goal of our exploratory study is to determine whether the information provided by news reports can increase knowledge about defensive gun use. We are interested in the types of incidents reported on, the information typically provided, and whether news reports might provide insights on current issues such as the incidence of self-defense gun use with assault rifles. As we did with homicides by children (Hemenway and Solnick 2017), unintentional gun use (Solnick and Hemenway 2019), and firearm homicides by police (Wertz et al. 2020), we also try to provide a useful typology for the classification of defensive gun use incidents.

## Methods

We examined the news reports assembled by the Gun Violence Archive classified as “Defensive Use” for 2019 (the last full year before the pandemic). After reading 50 cases, we created a data collection form in Qualtrics with about three dozen response items, such as the state, month, and time of day of the incident; whether the incident occurred indoors or outdoors; whether the perpetrator had a gun; the relationship between victim and perpetrator; whether the defender was a security guard; any illegal drug involvement; the age, sex, deaths, and non-fatal wounds of victims and perpetrators; whether the defender was a victim or good Samaritan; and if the defender’s action led to the arrest of the perpetrator. One variable was subjective (“Is it difficult to determine who was the criminal and who was the victim?”). Table 1 provides the response items and the percentage of cases where the requisite information was available (“completion rate”).

**Table 1:** Coded variables, percent completed (information was available from the news reports), percent yes for completed variable, percent yes for all 418 cases

Variable	Completion rate (418 cases) (%)	% Yes in completed cases (%)	% Yes in all 418 cases (%)
US state	100		
Month	100		
Exact time	79		
Did the incident take place inside	88	51	45
Did the incident take place at the victim’s home	94	58	55
Was there a female defender	98	10	10
Was there a female perpetrator	99	9	9
Did the perpetrator have a gun	78	62	48
If the perpetrator had a gun, did they fire it	82	68	56
Did the defender fire any shots	99	90	90
Did the defender fire more than one shot	56	65	36
Did the defender fire any warning shots	54	10	5
Any victim fatalities	100	5	5
Any perpetrator fatalities	100	35	35
Was the defenders gun a handgun	48	91	44
Where was the defender’s gun*	88		
In their home		47	41
On their person		36	32
At their workplace		8	7
In their car		5	4
Relationship between perps and victims*	72		
Strangers		39	28
Acquaintance		19	14
Romantic		15	11
Family		9	6
The defender, a security guard/off-duty police	72	9	6
Did the incident occur in the daytime	86	31	27
Were illegal drugs involved	64	11	7
Was alcohol involved	54	10	5
Was it difficult to determine criminal vs victim	99	19	19
Any female victims	98	26	26
Was the defender arrested	97	9	9
If arrested, was the defender charged	77	56	43
Was the perpetrator arrested	99	42	42
If arrested, was the perpetrator charged	74	98	73
Did the defenders action (help) lead to the arrest of the perpetrator	99	31	31

*Only the 4 most common places or relationships are listed in descending order

Because of the time involved in reading the 1–5 news articles linked to each incident and filling out the data collection form, we decided to read only a representative sample of cases. To ensure that all seasons were represented, we randomly picked a month and then sampled all cases in 3-month intervals (January, April, July, and October). Three states (DE, ND, and RI) and the District of Columbia had 1–4 cases, but none in those 4 months. We sampled one case from each of these four places (the specific one determined randomly by a die toss) to try to ensure that we had cases from every possible state.

After reading 300 cases, we decided on a mutually exclusive typology of incidents and went back and added those codes for the 300 cases and for subsequent ones.

To ensure a level of inter-rater consistency, one of us (DH) re-coded a dozen cases already coded by each of the other two authors and compared the results. For all the results presented in this paper, the ratings were never lower than 90% identical for any case or any variable, and in none of the two dozen cases was there a difference in the coded typology category. We decided that there was enough consistency that it was not necessary for two raters to read every case.

## Results

The GVA reported 1597 defensive gun use incidents from the more than 7,500 news and police sources culled for 2019. We coded 434 individual incidents (27%). Police responded to and aided in the reporting of virtually every incident.

Although most incidents had more than one news story, many important details of the incident were often missing. The news stories usually reflected the story told by the victim, as interpreted and relayed by the police. The version of the perpetrator, neighbors, or bystanders were less often reported. The age and name of the perpetrator—if they died or were arrested—were typically reported. The age and name of the victims and defenders (the person who used a gun against the perpetrator) were virtually never given.

Of the 434 incidents we read, in 16 cases the defender did not appear to use a firearm. We excluded these 16 cases from our analyses, leaving 418 incidents.

Whether the incident occurred inside or outside could be determined in 88% of 418 defensive use incidents, and half the time it was inside and half outside (Table 1). Over half of the 418 incidents took place in or around the home of one of the victims. A female was one of the perpetrators in 9% of the incidents, and a defender in 10%.

Of the 418 incidents, in 48% the perpetrator was reported to be armed with a firearm; in 22% of cases, it was unclear. In over half the incidents in which the perpetrator was known to have a gun, they fired the weapon. In 90% of the defensive use incidents, the defender fired a shot—and fired more than one shot in more than a third of incidents (36%). In 5% of the 418 incidents, the defender was known to have fired a warning shot.

In the 418 incidents, 105 victims were shot and 26 of them died from their wounds (Table 2); the 26 deaths occurred in 22 incidents (5% of all incidents). In the 418 incidents, 315 perpetrators were shot and 152 of them died from their wounds; one or more perpetrator died in 35% of all incidents. The mean number of total people shot per incident was 1.00; the mean number of perpetrators shot was 0.75 and the mean number of victims shot was 0.25.

**Table 2:** Total number of victims and perpetrators shot, killed in the 418 cases

	Shot	Died from wounds	Case fatality rate (%)
Victims	105	26	25
Perpetrators	315	152	48
Total	420	178	42

The defender—the person who used a gun against the perpetrator—was almost always one of the victims. In 7% of incidents, the defender could be described as a good Samaritan (not shown).

There was only one case in which a defender was reported to have used an assault rifle—a case in which five young armed men, known to the victim, broke through his front door at 1 AM. He was ready with a fully loaded AK-47 and killed three of them.

We created a typology with 13 categories of incidents. To make the categories mutually exclusive, we used a hierarchical ordering listed below—an incident could only be in one of the ordered categories and the first one it was placed in eliminated the possibility it could be in any subsequent one.

1. The victim uses someone else’s gun—almost always the perpetrator’s gun. 2% of incidents
2. Defender is a security guard or off-duty police officer. 6% of incidents
3. Drug-related. 4% of incidents
4. Gang-like combat. 6% of incidents. The combatants seem to know each other, and shoot-outs typically occur.
5. Romantic partner disputes. 11% of incidents
6. Other family issues. 6% of incidents
7. Escalating arguments. 13% of incidents
8. Store robberies. 9% of incidents
9. Street robberies. 5% of incidents
10. Unoccupied vehicle theft. 5% of incidents
11. Unarmed burglary. Described as a burglary in the news articles or the perpetrator charged as committing a burglary rather than a robbery or home invasion. 7% of incidents
12. Home invasions. 20% of incidents. A little over half the time the perpetrator breaks in or the victim finds an armed perpetrator inside the home.
13. Miscellaneous. 6% of incidents

In creating the typology, we had these facts in mind. Currently, most people who obtain and own guns do so for self-defense, usually self-defense in the home and against a stranger (Azrael et al. 2017; Miller et al. 2022). We were thus most interested in defensive gun use at home against a likely stranger, especially during an assault or robbery.

We made using someone else’s gun the first category—the victim was not using their own gun, and in virtually every one of these cases, the perpetrator’s gun. Gun use by police or a security guard is almost always eliminated in self-defense gun surveys (Rand 2018). Many of the other self-defense categories appear to be targeted, with the victim known to be at higher risk, attacked by someone they know (e.g., drug-related, gang-like combat, romantic partner disputes, other family issues). Escalating arguments have been previously judged to be the least clear about who is the victim and who the perpetrator (Hemenway et al. 2000). Store robberies and street robberies are not at home. And unoccupied vehicle theft and unarmed burglary are closer to, but still might be considered distinct from home invasions.

The penultimate mutually exclusive category was home invasions; these included all remaining home incidents whether or not they appeared to be targeted or whether the victims and perpetrators seemed to know each other—both of which were often unclear. The final category was for miscellaneous cases. We initially had an additional category—sexual assaults—but with only three incidents we decided to place these incidents into the miscellaneous category. Table 3 provides examples of incidents that the three authors decided were representative of each category. Table 4 provides, by category, the percentage of all incidents, the percentage of deaths, and the likelihood for the perpetrator to be armed with a gun.

**Table 3:** Typology of incidents, with representative examples

1. The victim uses someone else’s gun—almost always the perpetrator’s gun. 2% of incidents
• Two teenagers, known to the residents, demand money. The older teen tries to shoot his gun, which jams. His gun is wrestled away and one of the residents shoots him 3 times with it, killing him. The younger perpetrator is held at gunpoint for the police
• A teen tries to rob a stand selling holiday items. When he puts his gun down to pick up the cash that the employees put on the counter, one grabs the perpetrator’s gun and shoots him in the face
2. Defender is a security guard or off-duty police officer. 6% of incidents
• At 2 AM outside an apartment complex, a woman has been robbed by 3 men. A security guard follows them and gunfire is exchanged. The guard holds all 3 men for the police
• Early in the morning, 2 armed masked suspects enter an all-night restaurant to rob it. Seeing a security guard, they shoot and wound him. He fires back but they flee
3. Drug-related. 4% of incidents
• The victim is shot when he does not pay for the illegal marijuana he is purchasing. He fires back but hits no one and is charged with illegal gun possession
• In a parking lot, two young men display handguns to rob a young man during a drug deal. The dealer grabs his own gun and shoots both men, killing one. The two perpetrators had committed another armed robbery that day
4. Gang-like combat. 6% of incidents. The combatants seem to know each other, and shoot-outs typically occur
• In the afternoon, in what news reports call a drive-by shooting, four men in a car shoot and wound one young man standing with a group of men. The others in the group fire back and the car drives away
• In the early morning at a gas station, 3 young men are in an expensive car. Another car pulls up with 3 young men in it and they try to rob the first group. Someone in the expensive car shoots one of the perpetrators, and both cars speed away
5. Romantic partner disputes. 11% of incidents
• Two men shoot each other over a woman. The victim is shot in the face, the perpetrator is shot in the leg
• A man arrives at the home of his former wife. He hits her male friend with a baseball bat. The male victim shoots and kills the ex-husband
6. Other family issues. 6% of incidents
• In the afternoon, a young man pushes his way into the home of his grandparents. An altercation occurs over money, and the grandfather shoots and kills the grandson. The grandfather is arrested
• Early in the morning, a young man is choking his mother and is shot and killed by his 60-year-old father. The father is arrested
7. Escalating arguments. 13% of incidents
• A fight between neighbors over loud music ends with one of them dead. In an apartment complex, a couple upstairs yells at a couple in a car to keep their music down. Later that day, when the car owner discovers that his car has been vandalized, he races upstairs to confront the person who had yelled at him. He opens the apartment door and is shot dead
• Saturday near midnight, two men are arguing with each other outside a residence. One pulls a gun, both men fire at each other, and one is wounded
8. Store robberies. 9% of incidents
• In the late afternoon, at a fast-food restaurant, an armed robber fires warning shots and demands money. A nearby store owner hears the shots, goes to the scene, and confronts the robber as he is leaving. A shootout occurs, no one is wounded, and the robber gets away with the money
• A young armed male tries to rob a store and is shot three times by an employee. The wounded perpetrator is arrested at the store
9. Street robberies. 5% of incidents
• In the early morning, a young man shoots into a group of teens he says were chasing him. A wounded teen is subsequently brought to a hospital by friends who say he was shot in a drive-by
• Shortly after 10 PM, a 27-year-old male says that a young man tried to rob him and he shot the robber many times and killed him, but not before the robber shot and wounded him. Residents called 911. The perpetrator may have also robbed another person that night
10. Unoccupied vehicle theft. 5% of incidents
• Around 3 AM, a man at home awakens and sees people entering vehicles in his driveway. He goes outside and shoots at them. They shoot back, no one is injured, and they leave
• A man hears a commotion outside his home and sees two men and his truck hood open. He fires a warning shot into the grass and they flee
11. Unarmed burglary. Described as a burglary in the news articles or the perpetrator charged as committing a burglary rather than a robbery or home invasion. 7% of incidents
• A man’s home has been burglarized in the past. In the early morning, his dog is barking wildly. He finds a burglar in his garage. His surveillance camera shows him firing warning shots, and the burglar runs away
• At 3 AM an unarmed burglar breaks into a business building by coming through the roof. He is shot and captured by the owner who happens to be living there
12. Home invasions. 20% of incidents. A little over half the time the perpetrator breaks in or the victim finds an armed perpetrator inside the home
• At 2 AM a 37-year-old starts banging on the back door. An 86-year-old resident grabs his shotgun, fires a warning shot, and then wounds the perpetrator in the torso when he continues to try to get in
• A little before sunset, an 18-year-old kicks the door down. The homeowner shoots him and calls the police. The teen flees, goes to the hospital and dies
• Around 2 AM, an armed 27-year-old tries to enter a home and is shot and killed by the resident (no other information available)
• A male in his late 60s shoots and kills a man in his early 40s he saw on his outside balcony
• Shortly before sunrise, the next-door neighbor in his 40s, armed with a machete, breaks windows to get into the home and is shot and killed
• Around 11 PM, two men knock on the door and enter an apartment with guns drawn in an attempt to rob the occupants. A woman resident kills one of them and the other flees
13. Miscellaneous. 6% of incidents
• A 30-year-old male tries to cash a fraudulent check. Police arrive at the bank, but the man has left. Police see him and chase him. Running away, he jumps into the backyard of a homeowner who has a firearm. The homeowner shoots at him but misses. He eventually is caught by police after a long chase
• In the morning, a teen pushing a lawnmower asks a man in his 70s if he can borrow his cell phone. The teen pretends to make a call and then runs away with the phone. A neighbor with a gun gets into his vehicle, finds the teen and gets the phone back. The teen runs away
• A man in his late 60s goes to the police with this story. He picked up an unknown woman and rejects her prostitution offer. They get out of the car and she tries to take his wallet, an altercation occurs, and people come to the aid of the woman. The man draws his gun for protection, then puts it in his back pocket, whereupon a male with an umbrella who had come to the aid of the woman steals the gun
• A road rage incident ends with the two drivers stopping, getting out of their cars and arguing in front of an unrelated person’s residence. The resident comes out with a gun and tells them to leave. They do so, but the resident says one motorist tried to run him over, so he shoots at the vehicle multiple times, wounding the driver

**Table 4:** By type of defensive gun use, percent of total incidents, percent of total deaths, and percent of incidents in which the perpetrator was definitely armed with a gun

Type	% of all incidents	% perp definitely armed w/gun	% of all deaths
Uses perp’s gun	2	100	2
Security guard	6	61	7
Drug-related	4	88	6
Gang-like	6	89	7
Romantic partner disputes	11	45	9
Other family issues	6	35	7
Escalating arguments	13	44	18
Store robberies	9	64	6
Street robberies	5	61	5
Unoccupied vehicle theft	5	22	3
Unarmed burglary	7	0	4
Home invasions	20	46	20
Miscellaneous	6	32	6
Total	100	48	100

## Discussion

Our core question was “what might be learned about self-defense gun use from news reports?” We used the incidents assembled by the Gun Violence Archive (GVA), which appear to be a largely comprehensive compilation of news stories about gun incidents. The GVA assembles news articles from some 7,500 daily sources, including some police reports (though virtually every article we read also had a link to one or more news reports).

### Size and scope

The GVA classifies incidents into categories such as mass shootings, unintentional shootings, “defensive use”, and officer-involved incidents. We found a couple of duplicate defensive use incidents in the GVA, and we determined that 16 of the 434 incidents that we did code did not qualify as a defensive gun use (i.e., the defender did not use a gun) for a false positive rate of 3.7%. We did not examine the possibility of false negatives that the GVA did not correctly classify every potential “defensive use” incident.

Compared to the other main sources of defensive gun use data—private surveys and the National Crime Victimization Survey (NCVS)—the GVA provides a larger sample of defensive gun uses: 1,597 in 2019. By contrast, in a 5-year period, the NCVS provided only 127 incidents (Hemenway and Solnick 2015), and the larger individual private surveys typically provide hundreds of cases. In addition, compared to surveys, the news reports almost always concern actual incidents that the police know happened—with dead bodies, bullets, witnesses—rather than claimed events.

We believe the GVA collects a high percentage of the universe of all gun fatalities from defensive gun use. Validation studies show that the GVA not only obtains news articles for 94% of all police firearm killing of civilians in the USA (Conner et al. 2019) but also for a high percentage of fatal interpersonal urban shootings (Kaufman et al. 2020). In the 418 defensive gun use incidents we analyzed, there were 147 incidents with a shooting death of perpetrators (152 deaths). The 434 incidents coded (including the false positives) represent 27% of all defensive gun use incidents in the GVA, and if that is representative of the total GVA, we estimate the GVA news reports show 559 perpetrators killed in self-defense in 2019. The Uniform Crime Reports estimates that there were 386 justifiable homicides by private citizens in 2019. These two sources of data seem relatively consistent, given that many of the GVA homicides might not be considered justifiable (e.g., drug-related incidents, gang-like shootings, escalating arguments).

Having news reports of defensive gun use homicides only in the hundreds is one of the various indications that the claim of millions of self-defense gun uses each year is an overestimate (Hemenway 1997; Cook et al. 1997). If private surveys were accurately reporting millions of yearly defensive gun uses—with the perpetrator reportedly shot in 10% to 15% of the incidents—there should be many more annual newsworthy deaths than the 500+ found in the GVA.

The GVA included only 43% of the non-fatal woundings known to the police in three cities (Kaufman et al. 2020), so news reports probably miss more than half of the non-fatal woundings by defensive gun use—especially since some woundings may not be reported to police. If the news stories assembled by the GVA equally covered fatal and non-fatal self-defense shootings, then the case fatality rate for the shootings of perpetrators by defenders would be 48%, which seems much too high.

News reports undoubtedly miss most of the defensive gun uses in which no one is shot. Indeed, in about 90% of the GVA incidents the defender fired at least one shot. A major weakness of the GVA is that—outside of instances where someone dies—they are not random or representative of the universe of defensive gun uses. By contrast, the NCVS can provide a representative national sample of adult defense gun use during serious personal and property crimes (in which the respondent survived) and private surveys can add to that by providing a representative sample of self-reported defensive gun use in other situations. But given that private survey respondents do not all provide completely accurate answers, the major problems with extrapolating from private surveys—forward telescoping and the well-known epidemiological problem of false positives for rare events—make any extrapolated national estimates seriously bias (Hemenway 1997).

### Circumstances

Compared to the NCVS and private self-defense gun surveys, news reports do not have a list of specific questions (e.g., where was the gun when the defender initially needed it? was the gun used a handgun?), though such information is often reported. Indeed, it would be useful for research if reporters were provided with a short list of identical questions/facts that many might be willing to try to find the answers to for any shooting.

Compared to the survey data, a great advantage of news reports is that they usually provide a verbal description of the event, at least from the perspective of the defender. Few private surveys of self-defense gun use ask the respondent for even a brief synopsis of the incident in their own words, yet such descriptions often provide a very different picture of events than given by the response to scores of yes/no questions (Hemenway et al. 2000; Hemenway and Azrael 2000).

Still, most of the news articles also leave much to the imagination. Reading these incidents, we almost always wanted to know more—particularly the backstory, such as the reasons for the escalating arguments and why particular homes were picked for home invasion. But the news stories still typically provide much richer information than what is currently available from the NCVS and private surveys.

Another problem with the NCVS and private surveys is that they only provide the perspective of the defender and thus give an incomplete and potentially bias version of events. The news reports improve on this—a little. Almost all are filtered—a bit—by the police, who occasionally report discrepancies between the defender’s version and the physical evidence, or by the information provided by other relevant people. Sometimes, but not nearly enough, the news reports obtain information from the perpetrator, witnesses, neighbors, or family. Such information adds to and can radically change the readers’ understanding of the events.

The news stories can also provide data rarely available from the NCVS or private surveys. For example, while we did not incorporate this information for the current article, the news reports typically provide the exact location of the event—not just the state and town, but also the precise street address. The news stories also provide the age and full name of the perpetrator—at least if they die or are apprehended. However, the news stories rarely provide either the age or name of the defender.

We also believe that the news reports can help create a useful typology of events—which was one of the main goals of our study. Categories are important for understanding the events and for devising effective policies and programs.

Note that our making the categories mutually exclusive meant that each incident was placed into only one category—the first one of the 13 categories into which it fit. Making categories mutually exclusive ensured that adding the percentages together from all categories would sum to 100%, and each incident was counted only once. But it also meant that while some of the security guard and drug-related incidents were street robberies, and some of the gang-like combat, romantic partner, and other family issues incidents were escalating arguments, they were not included in those latter categories. If we had made escalating arguments the first category instead of the 7th, that would have increased the percentage of incidents classified as escalating arguments and decreased somewhat the percentages of the first six categories.

### Impressions

Vivid news stories from the Gun Violence Archive provide for many impressions that might not arise from merely tabulating yes/no responses to a set of question about each incident. Here, we focus on one issue: whether the gun use was beneficial to society.

The RAND literature review found that evidence for a causal effect of defensive gun use on reducing harm to society was “inconclusive” (RAND 2018). One reason for their conclusion was that there was so little scientific evidence on the issue. Indeed, a good first step in this field of study might be to try to reach consensus concerning not only the precise definition of defensive gun use but the basic criteria to be used to determine its societal benefit or harm.

From a public health perspective, we thought the best outcomes were those where there was both a clear criminal perpetrator and innocent defender and the defender was successful in preventing injury to themselves, while minimizing harm to others, including the perpetrator. However, we decided not to code for whether the gun use was beneficial because there was usually not enough information to make an informed judgment. Most important, as in all previous self-defense studies, we could not determine the counterfactual—what would have happened without the defensive gun use.

One impression was immediate: The extreme idea that virtually all defensive gun uses are good for society is incorrect. The gun use by the *perpetrator* always seemed bad for society. But the opposite did not appear to be true: Gun use in self-defense did not always seem to be beneficial.

Since “pro gun” sources commonly present stories of purportedly beneficial defensive gun use [e.g., the NRA’s American Rifleman weekly provides “real stories of law-abiding citizens who used their firearms to save lives” (National Rifle Association 2022); see also Cramer and Burnett 2012], we present a few alternative examples from our sample of GVA cases.

In many cases, the defender did not look like an innocent. Cases, where the defender did not call 911 or was not around when the police arrived, often seemed especially suspicious. Indeed, in our subjective judgments, in about 19% of the reported incidents, it appeared to us that either both parties were engaging in illegal behavior (e.g., drug deals, gang-like violence), or it was difficult to distinguish the perpetrator from the victim (e.g., escalating arguments).

Gun use in escalating arguments typically provided little evidence of societal benefit, whether or not the gun use may have been understandable from the defender’s perspective. In one case, in the early morning a 36-year-old at a motel called management and the police, complaining about the loud noise in the motel room above him. The police came and left. The noise started up again, and, finally, around 3AM the man went upstairs and started banging on the door. The defender, in his early 20s, claimed the man was trying to get in, so he shot and killed him.

Without our hierarchical ordering, defensive gun use in many family issues might also be classified as escalating arguments. After his parental visit, a young father kept his one-year-old son and refused to give him back to the mother. When law enforcement did not rectify the situation, days later, in the late morning, she went with a group of friends to demand the child’s return. Unarmed she forced her way into the father’s home. He said he was afraid she would grab his gun, so he shot and killed her. The killing was considered legally justifiable.

In escalating arguments, brandishing a gun, even without firing, can be questionable. At a car dealer, a customer’s daughter test-drove a vehicle, and it ran out of gas. The father was irate; he arrived at the dealer yelling and cursing and refusing to leave. The dealer went into his office and got his gun.

In many cases, it was clear who was the potential perpetrator and who was the innocent defender, and it often seemed that it was beneficial for the defender (and potentially for society) that the defender had used their gun (examples are in Table 3). However, it often seemed that it might have been better for the defender and/or for society if the defender had not used the gun or had used it differently.

For example, a handful of home invasion cases involved an intoxicated, disoriented young male in the early morning hours trying to enter a home that he claimed was his own. In these defensive gun use stories, the best outcome was when he was held at gunpoint until the police arrived to straighten things out; the worst outcome—which sometimes occurred—was when he was shot and killed. Similarly, in many cases of unoccupied vehicle theft it did not seem that public health was promoted when unarmed teens were shot and sometimes killed.

In all the store robberies, it was usually clear who were the “bad guys”—the masked, armed, young men demanding money. But even here, gun use sometimes seemed more dangerous than beneficial. For example, after two men robbed a grocery store of $80, a clerk pulled out a gun and fired four shots at them. No one was hit, including, fortunately, the five young children in the store at the time. The robbers got away.

## Conclusion

News stories provide an incomplete and bias picture of defensive gun use, but so do the other sources of information currently available. News stories seem to report on most of the defensive gun use homicides, and they provide a story of the actual events. The stories show that there are different types of defensive gun use. From the stories, we created a typology of incidents, some of which generally appear far less likely to be socially beneficial than others.

## Data Availability

All data are available online to the public at the Gun Violence Archive Web site. https://www.gunviolencearchive.org.

## Abbreviations

GVA: Gun Violence Archive
NCVS: National Crime Victimization Survey
